# Size of Cells and Physicochemical Properties of Membranes are Related to Flavor Production during Sake Brewing in the Yeast *Saccharomyces cerevisiae*

**DOI:** 10.3390/membranes10120440

**Published:** 2020-12-18

**Authors:** Tsuyoshi Yoda, Tomoaki Saito

**Affiliations:** 1 Aomori Prefectural Industrial Technology Research Center, Hirosaki Industrial Research Institute, 1-1-8 Ougi-machi, Hirosaki, Aomori 036-8104, Japan; tomoaki_saito@aomori-itc.or.jp; 2 The United Graduate School of Agricultural Sciences, Iwate University, 3-18-8, Ueda, Morioka 020-8550, Japan

**Keywords:** lipid vesicles, ethyl caproate, isoamyl acetate, fluidity, yeast

## Abstract

Ethyl caproate (EC) and isoamyl acetate (IA) are key flavor components of sake. Recently, attempts have been made to increase the content of good flavor components, such as EC and IA, in sake brewing. However, the functions of EC and IA in yeast cells remain poorly understood. Therefore, we investigated the effects of EC and IA using cell-sized lipid vesicles. We also investigated lipid vesicles containing EC and/or caproic acid (CA) as well as IA and/or isoamyl alcohol (IAA). CA and IAA are precursors of EC and IA, respectively, and are important flavors in sake brewing. The size of a vesicle is influenced by flavor compounds and their precursors in a concentration-dependent manner. We aimed to establish the conditions in which the vesicles contained more flavors simultaneously and with different ratios. Interestingly, vesicles were largest in a mixture of 50% of 1,2-dioleoyl-sn-glycero-3-phosphocholine (DOPC) with 25% EC and 25% CA or a mixture of 50% DOPC with 25% IA and 25% IAA. The impact of flavor additives on membrane fluidity was also studied using Laurdan generalized polarization. During the production process, flavors may regulate the fluidity of lipid membranes.

## 1. Introduction

Ethyl caproate (EC) and isoamyl acetate (IA) contribute key flavors to fermented foods and beverages such as sake and juice. Although the functions of EC and IA in yeast cells remain poorly understood, efforts are being made to increase the content of good flavor components, such as EC and IA, in yeast [1,2,3,4]. As lipid vesicles have properties similar to cell membranes, artificial cell-sized lipid vesicles have been used as cell model membranes to reveal physicochemical properties of membranes that are important for life processes. Therefore, we have used a vesicle system formed by a single phospholipid component to study the physical—chemical effects of the flavor components [5]. Of note, good flavor components and their precursors influenced the behavior of the lipid vesicles, i.e., their size and fluidity, in a concentration-dependent manner. These results may be useful to better understand how flavor production in yeast affects the physicochemical properties and physiological function of cell membranes.

**Figure 1:**
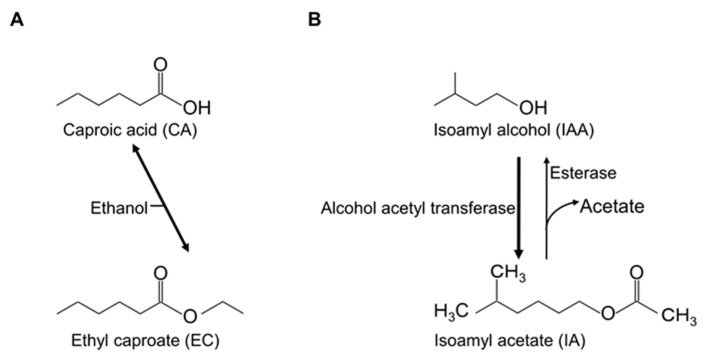
Structure and production pathway of key flavors. The production pathways of ethyl caproate (**A**) and isoamyl acetate (**B**).

Although good flavor components are well known to increase the quality of beverages and foods, little research has focused on their physiological functions. There is great interest in increasing the productivity of yeast strains, and EC productivity has been investigated using several techniques. A popular method involves analyzing fatty acid synthase mutations [1,6]. In the yeast cell, long-chain fatty acids are involved in membrane construction. EC is produced from the precursor caproic acid (CA) and ethanol via esterification (Figure 1). CA is mainly biosynthesized using acetyl-CoA and malonyl-CoA as substrates in the fatty acid synthesis pathway of sake yeast during brewing [1]. Therefore, CA and EC are considered to be produced as by-products of long-chain fatty acid synthesis in yeast cells. As such, fatty acid synthase mutations lead to the production of numerous short- and medium-length acyl chain molecules, such as CA, rather than long acyl chains. EC is then produced from CA and ethanol; however, usually actual EC productivity is confirmed by small-scale brewing. Both fatty acid synthase analysis and small-scale brewing require expensive equipment and a substantial amount of time.

The production pathway of IA is also known [7]. IA is obtained using 5,5,5-trifluoro-DL-leucine-resistant yeast strains with improved IA production [7,8]. IA is synthesized from isoamyl alcohol (IAA) by alcohol acyltransferase [9]. The precursor IAA is known for its bad flavor [7]. These flavors are shown in Figure 1.

Conventionally, contents of such flavors are measured by gas chromatography or headspace gas chromatography. Furthermore, stir-bar sorptive extraction methods with gas chromatography-mass spectrometry are used to measure off-flavors in beverages [10]. Although these methods offer great accuracy in measuring flavors, they also have disadvantages, including expensive machinery, running costs, and relatively long time periods. Alternatively, lipid vesicles have been used to examine functions of biological compounds and molecules. 1,2-dioleoyl-sn-glycero-3-phosphocholine (DOPC) has been used as an unsaturated lipid model for yeast membranes previously [11,12]. Vanegas et al. demonstrated an unsaturated lipid model system that, in comparison to previous studies, more closely resembles a real yeast plasma membrane [12]. In previous studies, we have also investigated the interaction between biological molecules, such as capsaicin [13], oxidized cholesterol [14,15,16], proteins [17,18,19], and polyphenols [20,21], and DOPC-containing lipid membrane vesicles.

**Figure 2:**
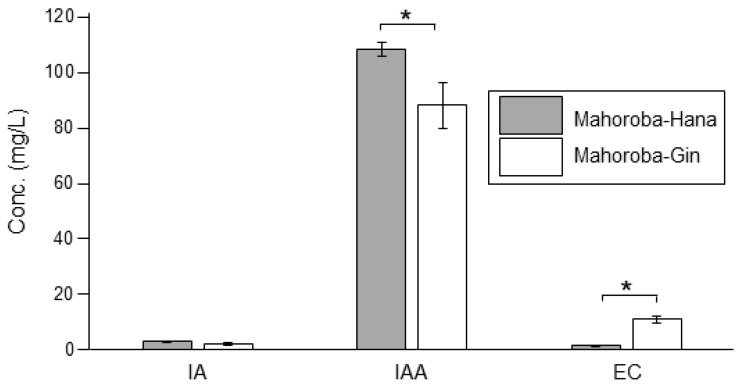
Flavor concentrations of sake prepared using the two strains, Mahoroba-Hana and Mahoroba-Gin. Isoamyl acetate (IA), isoamyl alcohol (IAA), and ethyl caproate (EC) are shown. Gray refers to Mahoroba-Hana, and white illustrates Mahoroba-Gin. Average of four samples with standard error (* *p* < 0.05).

**Figure 3:**
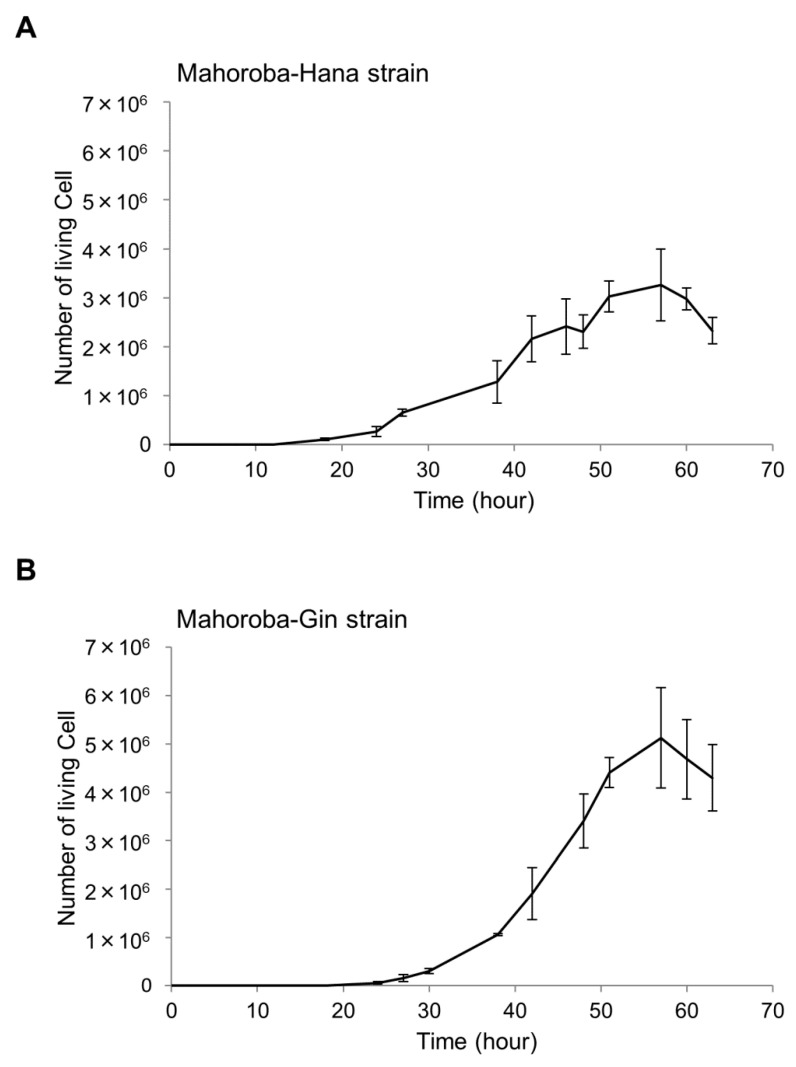
Growth curves of two yeast strains. The number of living cells was enumerated for the Mahoroba-Hana (**A**) and Mahoroba-Gin strains (**B**).

Recently, we produced lipid vesicles containing EC and its precursor CA and found that these vesicles were small [5]. The diameter of vesicles affects several physicochemical properties [22,23]. According to previous research, EC and CA should interact with lipid membranes [5]. We have postulated that different membrane properties correspond to different flavors; this could enable rapid detection and quantification of flavors in the future. Herein, we report lipid vesicles containing IA or IAA. The different vesicle diameters corresponded to different IA and IAA concentrations. This could be applied to detect different flavors in alcoholic beverages such as sake. Furthermore, the diameters of vesicles that could be considered to produce flavors during sake brewing were investigated. Although trends regarding the correlation between size and flavor were observed, an unexpected result was also found that the vesicle size was largest at equal amount of flavor and precursor concentrations though the trends indicated that vesicle diameter increased with increasing flavor concentrations and decreasing precursor concentrations. Therefore, Laurdan generalized polarization (GP) was performed for the vesicle membranes containing flavors and their precursors, as this measurement reveals the degree of hydrophobic carbon chain order of phospholipid molecules in membranes [24,25,26]. Our institute has developed the yeast strain *Saccharomyces cerevisiae*, Mahoroba-Gin, which exhibits high EC productivity, and Mhoroba-Hana [27,28]. These strains can be obtained from our institute, and a fee payment is required for the same. With this strain, sake contains ~10–12 ppm of EC, whereas one sake production company has reported that sake produced using normal yeast strain contains 1.4 ppm of EC [29]. Our institute prepared sake using the two strains Mahoroba-Gin and Mahoroba-Hana (normal strain, and a parent strain of Mahoroba-Gin) as test brewing, and the data on flavor concentration of these sake preparations are shown (Figure 2). The production of EC of Mahoroba-Gin was significantly higher for that of Mahoroba-Hana (Student’s t-test, *p* < 0.05) (Figure 2), their IAA concentrations were significantly different. The EC concentration of the sake made with the Mahoroba-Gin strains was seven times higher than that with the Mahoroba-Hana strains (Student’s t-test, *p* < 0.05) (Figure 2). Of note, CA concentration data is lacking as the measurement of CA and EC concentrations at the same time was difficult. Therefore, our established strain is considered a highly productive strain compared with normal yeast strains. The growth curves and cell diameters have been reported before [5] and are now discussed in relation to the present data (Figure 3).

## 2. Materials and Methods

### 2.1. Materials

1,2-Dioleoyl-sn-glycero-3-phosphocholine (DOPC) and IAA were purchased from Tokyo Chemical Industry Co Ltd. (Tokyo, Japan). Ultrapure water obtained from Millipore Milli-Q purification system (Millipore, Bedford, MA, USA) was used for reagent preparation and glassware cleaning. EC, IA, acetone, ammonium sulfate, methanol, sodium chloride and glucose were purchased from Wako Pure Chemical (Osaka, Japan). Chloroform was purchased from Kanto-chemical (Tokyo, Japan). CA and yeast nitrogen-based medium without glucose or ammonium sulfate were obtained from Aldrich (St. Louis, MO, USA), Kanto-Chemical, and Difco Laboratories (Detroit, MI, USA).

### 2.2. Preparation of Lipid Vesicles

Acetone was used as washing solution for glass test tubes. Several different types of lipid vesicles (giant unilamellar vesicles and model membranes/liposomes) were prepared. A slightly modified version of the method of natural swelling from dry lipid films was used as outlined in our previous research [5,14,15,16]. Mixtures of lipids and flavors (i.e., DOPC, EC, CA, IA, and IAA) were dissolved in chloroform in a glass test tube under nitrogen gas. The glass test tubes were already washed with acetone and dried by a draft. Next, they were dried under vacuum for 3 h to form thin lipid films. Then, the films were hydrated overnight with ultrapure water at room temperature (20 °C). The final concentration of the hydrated film was 0.2 mM of lipid. The formations of unilamellar vesicles are highly dependent on the circumstance of preparation. Therefore, we carefully prepared the samples and adjusted the conditions as described below. The thin lipid films were kept in a vacuum before hydration with water. During hydration, the test tube was double-wrapped with parafilm and aluminum foil to prevent oxidation. The test tube was stored in a drawer at a constant temperature in the dark until before observation. The observation would take place within a week.

### 2.3. Microscopic Observation

The lipid vesicle solution (6 μL) prepared as per abovementioned method was placed in silicon wells (0.2 mm) on a glass slide and covered with a small cover slip. Trends of diameter in lipid vesicles were then observed using phase-contrast microscope (BX53, Olympus, Japan, [5]) at room temperature. The types and sizes of the lipid vesicles were investigated. At least 30 lipid vesicles were observed for each type. Lipid vesicles were chosen randomly, and their sizes were measured in diameter. To avoid bias and confirm the results, some experiments were repeated by another person. Lipid vesicles were prepared at least three times, and it was confirmed that there was no large bias in each preparation.

### 2.4. Yeast Cell Growth

Slant yeast was stored in a refrigerator at 5.0 °C. We plated the slant yeast in autoclaved yeast nitrogen-based medium, as used in seed yeast. First, we diluted the seed yeast at 30 °C at a concentration of 1 × 10^5^ cells. Cell counting was performed using cello-meter (Nexcelom Bioscience LLC, Lawrence, MA, USA) with attached fluorescence reagents (yeast dilution buffer and yeast live dead staining solution) [30]. We slightly modified the final diluted ratio to 6–20-fold of that of the yeast medium. The fluorescence light exposure time was set to 600 ms. According to the growth curve (Figure 3), we determined the phases of both Mahoroba-Hana (lag phase: 18 h, log phase 38 h, and stationary phase: 51 h) and Mahoroba-Gin (lag phase: 27 h, log phase: 48 h, and stationary phase: 57 h) to measure the cell sizes.

### 2.5. Measurements of Membrane Fluidity

The fluidity of membranes containing DOPC and flavors was measured using excitation Laurdan GP [13,24,25,26]. The Laurdan fluorescent label was used at 1% (*v*/*v*) concentration. The final lipid concentration was 0.2 mM. Liposomes were observed at 410 and 505 nm (Laurdan emission) using a dichroic mirror of a fluorescence microscope (Olympus BX53 with attached fluorescence unit, Olympus Japan). The Laurdan GP value was defined as GP = (I(420–460) − I(510–550))/(I(420–460) + I(510–550)), where I(420–460) and I(510–550) are the average fluorescence intensities of Laurdan detected at ranges of 420–460 and 510–550 nm, respectively.

### 2.6. The Detection of Flavor Compounds in Lipid Vesicles by Gas Chromatography-Mass Spectrometry

Gas chromatography-mass spectrometry (GC/MS) with a 7890B GC System/5977A MSD (Agilent Technologies, Inc., Santa Clara, CA, USA) and 5 μm filters (Millipore, County Cork, Ireland) was used to determine the presence of IA, IAA, or CA in lipid vesicles. The chemical 3-octanol was added as the internal standard to the lipid vesicle solution with 50% of IA, IAA, or CA, and sodium chloride was measured using stir-bar sorptive extraction [5]. To prevent flavors presence out of the membranes, the filter was caught because it is not possible to simply attach a filter even if the flavor compounds are present in the solution before they reach the membranes. As a comparison, we made a solution by mixing flavors and DOPC. When preparing these solutions, sodium chloride was added to promote extraction [5]. Each experiment was conducted three times.

### 2.7. Statistical Analysis

Image processing was performed using ImageJ software (https://imagej.nih.gov/ij/download.html). Data are expressed as mean ± standard error of 3, 15, or 30 independent experiments. Student’s t-test, two-way ANOVA, and F-test in Excel were used to analyze the statistical significance of the differences. The vesicle dispersions exhibit Gaussian distribution profiles. Therefore, in the Figures, the diameter of the vesicles was represented by the average size and standard error.

## 3. Results and Discussion

### 3.1. Diameters of Flavor-Containing Lipid Vesicles

**Figure 4:**
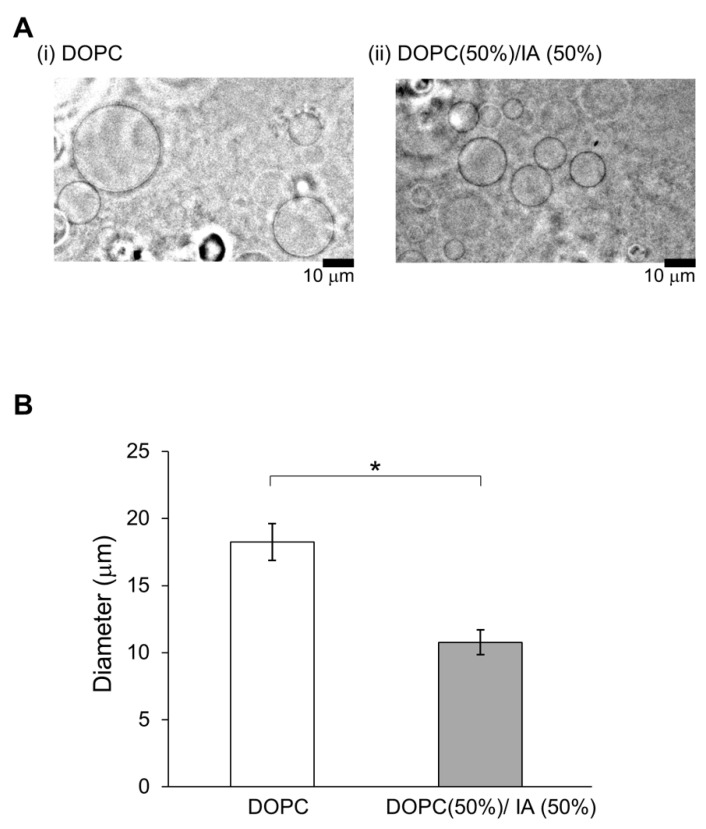
Vesicles containing isoamyl acetate (IA) are smaller. (**A**) Images of typical lipid vesicles captured using a phase-contrast microscope: (**i**) DOPC and (**ii**) DOPC (50%) with IA (50%). Scale bars: 10 μm. (**B**) Vesicle diameter based on contents: vesicles containing DOPC (white) and vesicles containing 50% DOPC and 50% IA. (* *p* < 0.05).

**Figure 5:**
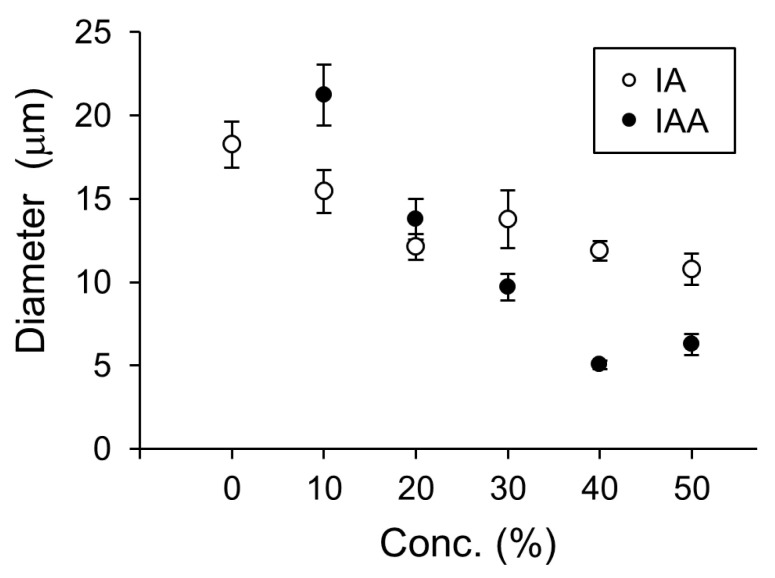
Diameters of vesicles containing isoamyl acetate (IA) or isoamyl alcohol (IAA) at different concentrations (0–50%). Lipid vesicles containing isoamyl acetate (IA, white dots) and isoamyl alcohol (IAA, black dots).

**Figure 6:**
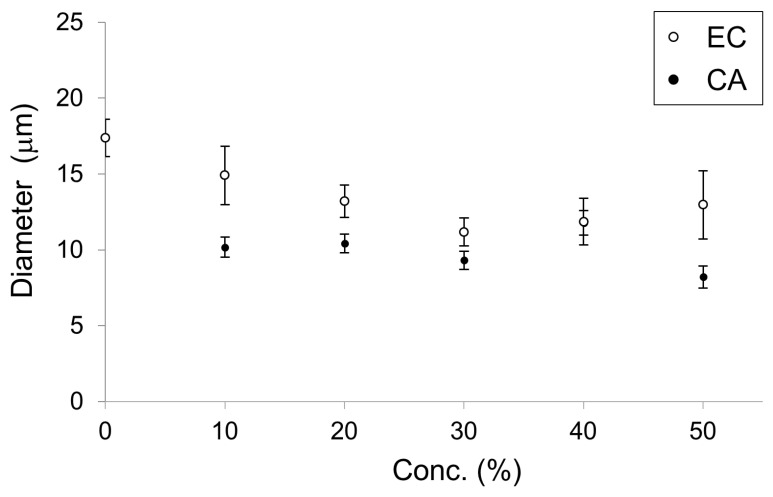
Diameter of lipid vesicles with membranes containing ethyl caproate (EC, white dots) and caproic acid (CA, black dots) at concentrations from 0 to 50%. These data were reconstructed from our previous study [5].

In a previous study, membrane dynamics of 50% DOPC/50% cholesterol were investigated under oxidative stress on the effect of containing of cholesterol [14] because 50% cholesterol is nearly the maximum content to form a cell-sized vesicle. Likewise, 50% was considered a better EC content to demonstrate the effect on physiological function [5]. Therefore, we intended to explore the effect on physiological function for IA on 10–50% relatively strong concentration, although the concentrations of the flavors in the in vitro experiment were not used under physiological conditions. The vesicles whose membranes contained DOPC and IA were significantly smaller than vesicles whose membranes contained only DOPC (Student’s t-test, *p* < 0.05) (Figure 4). Next, the effects of IA concentration on vesicle diameters were investigated. The smallest average diameters occurred at 50% IA. At IA concentrations of 10%, 20%, 30%, 40%, and 50%, vesicles were smaller than the vesicles without IA (>20% IA concentration) (Figure 5). Furthermore, the smallest average vesicle diameter occurred at 40% IAA (Figure 5). Although 10% IA and IAA did not affect vesicle diameter, > 30% IA and IAA affected vesicle diameter. IAA (>30%) exerted a significant greater effect on vesicle diameter decrease than IA (two-way ANOVA, *p* < 0.05). Regarding the vesicle diameter decrease at 10% IA and an increase at 10% IAA, statistical difference was not observed when compared with that of 0 containing. More than 30% IA and IAA significantly affected the vesicle diameter. More than 30% IAA exerted a greater diameter decrease than IA. Recently, we reported that a yeast strain producing high quantities of EC in its stationary phase decreased in diameter during EC production [5]. As CA is the esterification precursor of EC in yeast, we have compared vesicles containing CA versus EC (Figure 6). The precursor CA exerted a significant stronger effect on diameter reduction than EC (two-way ANOVA, *p* < 0.05). Interestingly, this trend was similar for IA and IAA. Similarity trends were observed indicating that precursors exhibit a stronger effect in decreasing the diameter of lipid vesicles rather than flavors. Almost same trends were found with a significant difference at >30% concentration of each good flavor and its precursor in totally without 40% of EC and CA.

**Figure 7:**
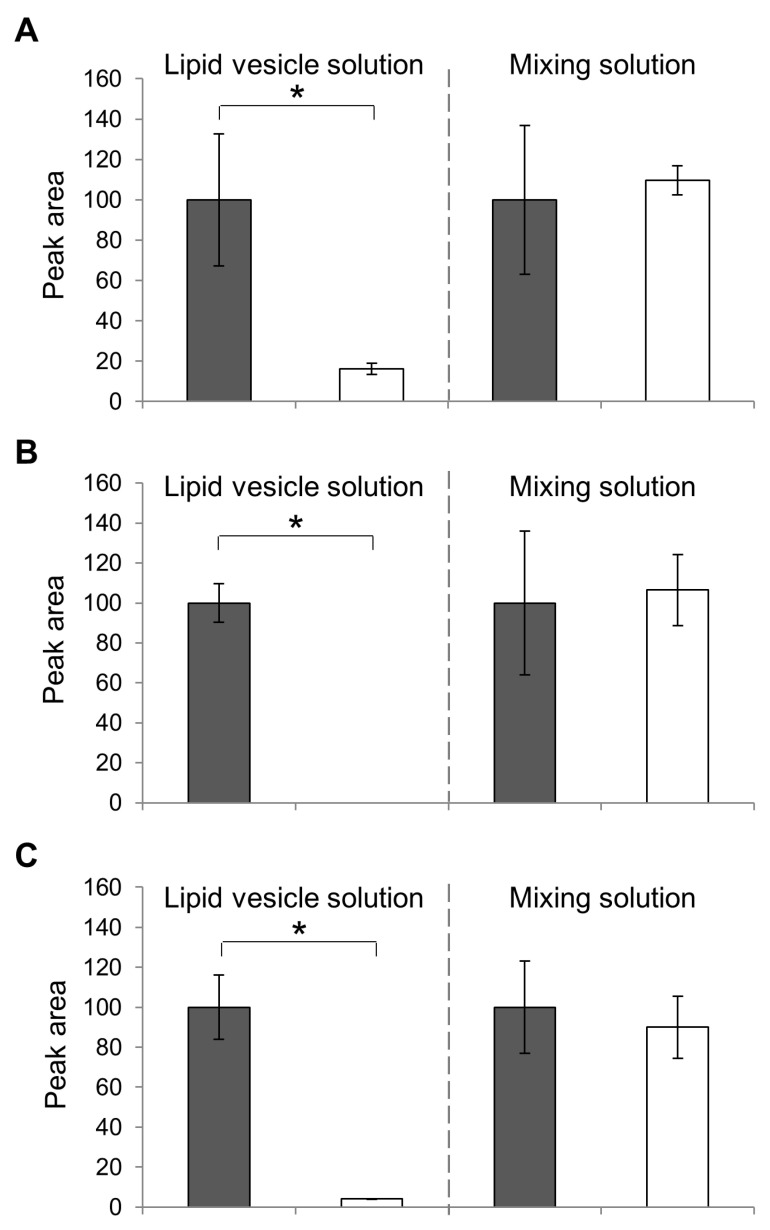
GC/MS spectrometry of IA (**A**), IAA (**B**), and CA (**C**) vesicles. The peak area of the pre-filtered (gray bars) and its filtered solution (white) solution of the lipid vesicle solution (left bar graph) and the mixed solutions (right) are compared. The peak values are calculated as a percentage of the pre-filtered solution (100%). Each bar represents the average value of three samples; standard errors are shown as error bars. (* *p* < 0.05).

Subsequently, the presence of flavor compounds in the membrane of the lipid vesicles was confirmed. Previously, we confirmed the presence of EC in lipid vesicles using GC/MS analysis and filtered solution [5]; also, the concentration of EC was reduced in the filtered solution compared to that in the pre-filtered solution. In addition, the presence of flavors IA, IAA, and CA in membranes was confirmed. In the present study, the value of the peak area of IA(A), IAA(B), and CA (C) was measured and compared (Figure 7). We confirmed that, at 16, 0, and 4.0, the peak area value of the filtered lipid vesicle solution of IA, IAA, and CA, respectively, showed a significant reduction compared to the corresponding pre-filtered solutions (F-test, *p* < 0.05). In the IAA detection experiment, the peak of IAA was not observed even though other peaks, such as the rest solvent chloroform and the internal standard 3-octanol, were observed. It is possible that the IAA molecules in the vesicles could not pass through the filter with smaller 5 μm pores and therefore, got stuck in the filter. They also had the same concentration of flavors in just mixing the solution with DOPC. Although when based on their character of flavors were caught filter it could not make sure the presence of flavors on lipid vesicles; these phenomena could not be observed. As a result, there was no significant difference between the detection of the flavor compounds in the filtered solution and the pre-filtered solution. These results suggested that flavor compounds were enclosed by the membrane of lipid vesicles, reducing the amount of flavor compounds caught by the filter. These results indicate that during the production of lipid vesicles with flavor compounds, the flavor compounds could be captured in membranes securely.

According to the results of the diameter of lipid vesicles, we believed that to develop DOPC lipid vesicles containing the flavor compounds or their precursors or both in an alcoholic beverage, the vesicle diameter may be used as the first screening criterion for these compounds, although actual beverages may contain unintended impurities. However, there are certain issues. For example, the compounds will not be able to be inserted into a lipid membrane. Therefore, we plan to generate lipid vesicles containing the flavor compounds, their precursors, or a mixture of both via hydration as a method to study alcoholic beverages in the future.

### 3.2. Diameter of Lipid Vesicles Containing Flavors and Precursor

**Figure 8:**
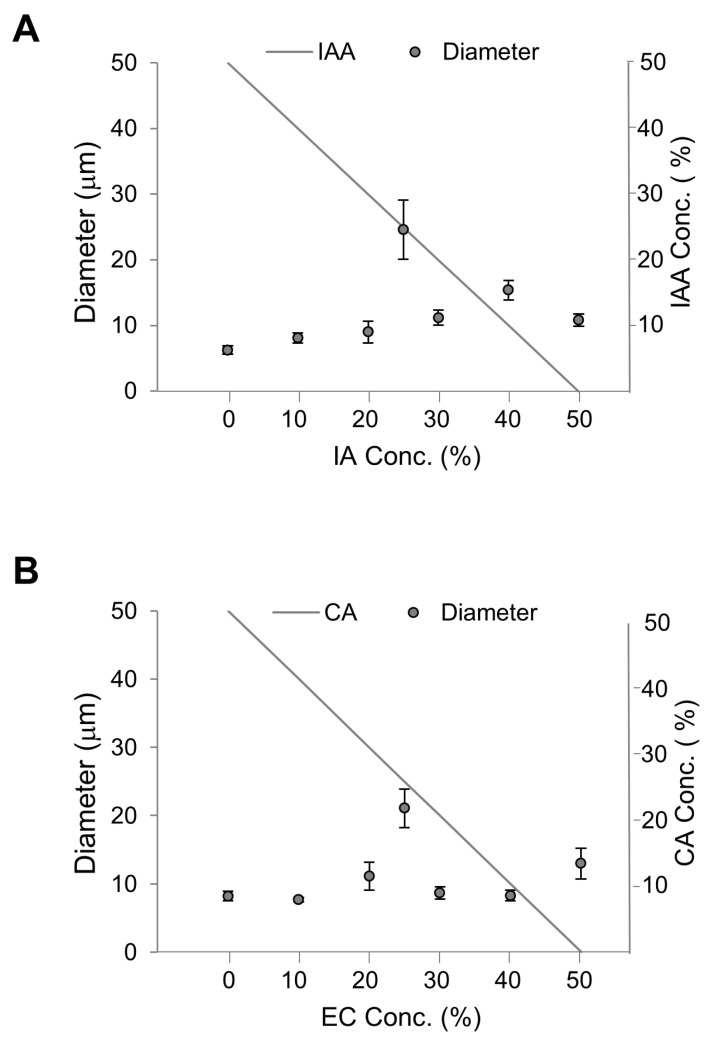
Diameters of vesicles containing key flavors. The total flavor concentration was constant at 50%, and the ratio of flavor-to-precursor was variable. (**A**) Diameters of vesicles containing isoamyl acetate (IA) and isoamyl alcohol (IAA). (**B**) Diameters of vesicles containing ethyl caproate (EC) and caproic acid (CA).

Next, we attempted to establish conditions in which the vesicles contained more flavors simultaneously and with different ratios. As 50% was considered optimal to investigate the effects of flavors on membranes, we produced vesicles containing 50% DOPC with EC and CA (Figure 8A). Each EC: CA ratio are (0:50), (10:40), (20:30), (25:25), (30:20), (40:10), and (50:0). Similarly, we produced vesicles containing 50% DOPC with IA and IAA (Figure 8B). Each IA: IAA ratio are (0:50), (10:40), (20:30), (25:25), (30:20), (40:10), and (50:0). A trend of increasing vesicle diameter was observed with increasing EC concentrations and decreasing CA concentrations. However, the vesicle diameter was significantly largest at 25% EC and 25% CA. Similarly, a trend of increasing vesicle diameter was observed with increasing IA concentrations and decreasing IAA concentrations, and the vesicle diameter was significantly largest at 25% IA and 25% IAA. Altogether, vesicles are largest when they contain 25% of each flavor. Referring to a previous study on large droplet formation from phospholipid membranes [31], we postulated that the vesicle diameter increase is a result of decreased fluidity and unstable lipid chain packing.

### 3.3. Membrane Fluidity of Lipid Vesicles

**Figure 9:**
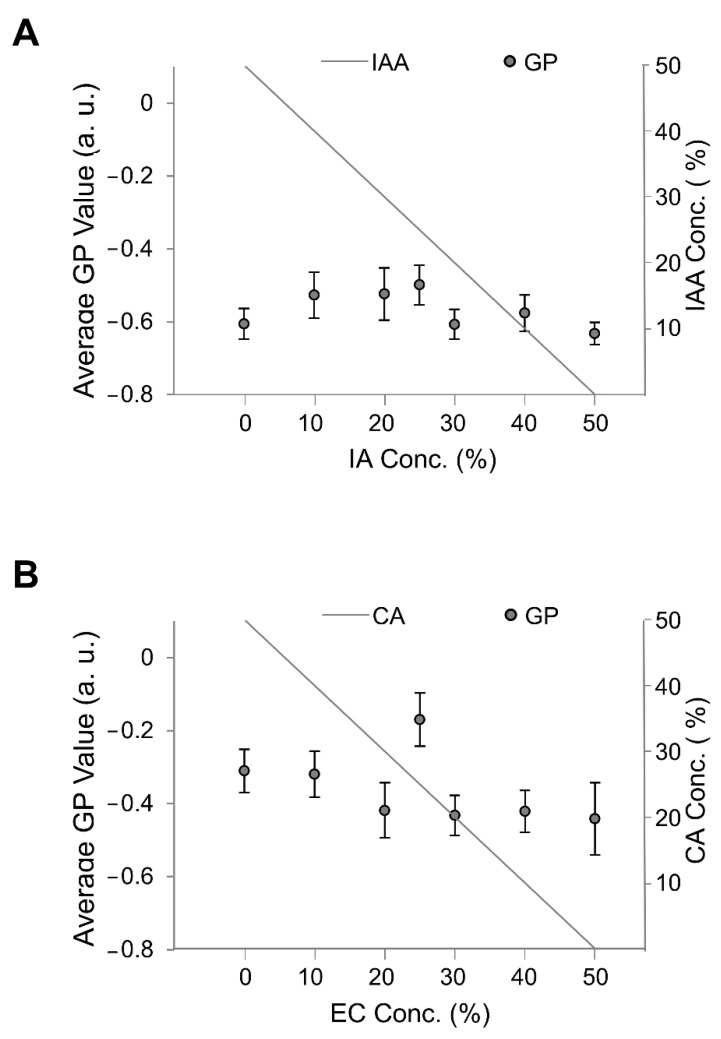
Average GP values of membranes containing different flavors. The total flavor concentration was constant at 50%, and the flavor-to-precursor ratio was variable. (**A**) Diameters of vesicles containing isoamyl acetate (IA) and isoamyl alcohol (IAA). (**B**) Diameters of vesicles containing ethyl caproate (EC) and caproic acid (CA).

Thus, we investigated membrane fluidity using membrane fluidity indicators, i.e., GP values, with the fluorescence probe Laurdan and fluorescence microscopy [13,24,25,26]. In our experiment, the Laurdan concentration was 1 mol% of the lipid concentration. The GP values of membranes containing DOPC with EC and CA and DOPC with IA and IAA are shown in Figure 8. Of note, the GP values were significantly largest for vesicles containing both 25% EC and 25% CA and 25% IA and 25% IAA though not significant compared with the other lipid vesicles containing IA and IAA. These results indicate that the large diameter of vesicles containing 25% EC and 25% CA is due to low fluidity even though GP is similar at vesicles containing 50% IAA and 50% IA. Likewise, it was the same for 50% IAA and 0% IAA. In the case of the IA and IAA system, the diameter of the vesicles (Figure 8A) increases almost steadily (with the outlier at 25/25%) with increasing IA concentration or decreasing IAA concentration; however, virtually no change in membrane fluidity was observed (GP values in Figure 9A), and there was no apparent correlation between membrane fluidity and vesicle diameter.

### 3.4. Membrane Fluidity and Cell Size

We further investigated how the size of lipid membrane vesicles would vary due to the physicochemical properties of the membrane, specifically, how the fluidity of the membrane affected the intermolecular interaction between the lipid molecule DOPC and added flavor compounds and their precursors. These results suggest that membranes with relatively low fluidity are in an unstable state that results in fusion and thus formation of large vesicles. The hypothesis is consistent with a previous study [31]. The article stated that saturated phospholipid distearoylphosphatidylcholine (DSPC) formed larger monolayer droplets than DOPC (unsaturated phospholipid). The article also mentioned that reducing energy cost for fusion and large droplet formation led to unstability of DSPC monolayer rather than that of DOPC. In addition, we have discussed that the diameter of lipid vesicles related to the shape of membrane molecules and their packing [5,21]. In the case of lipid vesicles containing the EC and CA system, the diameter of lipid vesicles might be regulated fluidity, although the IA and IAA system did not explain the fluidity of membranes. The diameter of lipid vesicles may be regulated by the balance of fluidity and packing of molecule on membranes. Strong molecular packing allows increasing the curvature, which reduces the diameter of lipid vesicles [5,21]; in contrast, strong molecular packing results in low fluidity of membranes, making them unstable to induce fusion chance to result in large size of lipid vesicles [31]. Therefore, the diameter of lipid vesicles might be determined by the balance of the two against interaction. Thus, vesicle diameter may be regulated by flavor content and physicochemical membrane properties, such as fluidity and molecular packing. Our method to make lipid vesicles is the natural swelling of a dried lipid film. The method could not control the layer in which the molecules were inserted. On the other hand, a droplet method [32] could allow compounds to be accurately into a specific layer or symmetrically or asymmetrically inserted in a membrane. We plan to apply this method to our research in the future because studying the factors that affect the localization of flavor compounds is significant and exciting.

**Figure 10:**
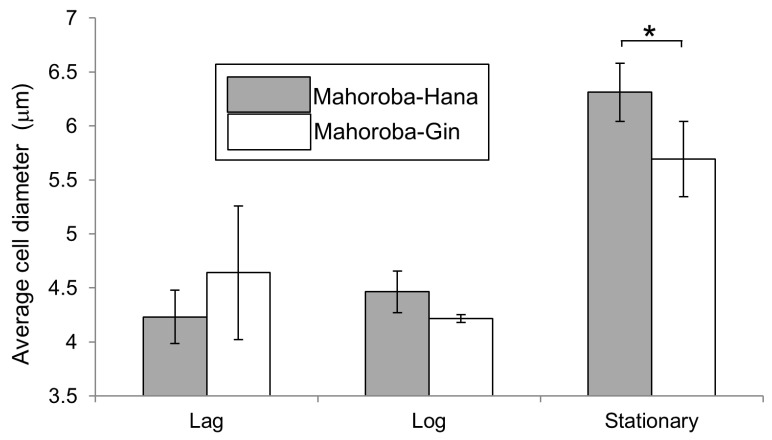
Average cell diameter at the lag, log, and stationary phases. Gray shows Mahoroba-Hana and white shows Mahoroba-Gin (* *p* < 0.05).

The fluidity of membranes is related to their physiological functions, such as responding to environmental stress, in various yeasts [33]. Our results enhance our understanding of membrane fluidity and life processes of yeast growth and flavor production. At the stationary phase, the yeast strain with high EC production (Mahoroba-Gin) exhibited smaller cell diameters rather than low productivity strain of EC (Mahoroba-Hana, Figure 10). Our institute prepared sake at a small scale as test brewing using the two types of strains, Mahoroba-Hana and Mahoroba-Gin. The flavor contents are shown in Figure 2. Sake was prepared using Mahoroba-Gin and Mahoroba-Hana, in which the concentration of IA does not differ significantly (Student’s t-test, *p* > 0.05) (Figure 2). Although the IAA concentration is also different in the two types of sake, the EC concentration of sake prepared using Mahoroba-Gin was seven times higher than that of sake prepared using Mahoroba-Hana (Student’s t-test, *p* < 0.05) (Figure 2). Although there were technical difficulties and CA concentrations were not measured, it has been reported that CA concentration corresponded to EC concentration in sake [34]. During sake brewing, the yeast growth progressed and their phase was stationary. Mahoroba-Gin should have EC and CA rather than Mahoroba-Hana in the stationary phase.

At the lag and log phases, the strains exhibit different diameters due to the changes in the quantities of flavors and their precursors, as this ratio influences membrane fluidity (Figure 9) and thus may affect cell diameter (Figure 8 and Figure 10). Furthermore, these results are useful for screening for yeast strains with good flavors, as the flavor-to-precursor ratio may be predicted from the size of yeast strains and membrane fluidity. In our previous study, we measured the cell diameter of yeast by microscopic observation [5]; in contrast, in the present study, we measured the cell diameter using an auto cell counter, and hence we believe that diameter difference could have occurred. Since in both measurements, the correlation between the size of yeast cells and the flavor-to-precursor ratios remain constant, we can use the yeast cells’ size. For measuring membrane fluidity, in the present study, we used membrane containing only the unsaturated lipid DOPC system. The DOPC membrane system is a single-component system and, therefore, easy to set up. It has the advantage of being highly reproducible if carried out carefully.

A cell membrane contains unsaturated lipids, saturated lipids, and sterol. Previously, a group reported that EC reduced a lipid-free cell wall’s ability to absorb contents, although the cell wall succeeded in absorbing a small number of flavor compounds [35]. The same authors reported that the binding capacity of yeast walls was not affected only by lipids. Our experiments and conditions were different from this study. In their experiment, EC was inserted between the cell walls in the model wine solution. In contrast, flavor compounds and lipids were prepared together to make membranes with different percentages of compounds and lipids. However, lipid vesicle systems have several disadvantages. Although the actual cells contain unsaturated lipids, saturated lipids, and sterol, the liposomes could not be formed at sterol conditions [33]. Subsequently, it has been known that the membrane components isolated from the yeast cell can be used to reconstruct vesicles with biomimetic cell membranes [33,36]. The system contains several kinds of lipids. We planned to add flavor compounds and their precursor to the membranes system to better understand their effects. We then planned to use more mimetic cell membranes containing those types of lipids and flavors. Although currently we do not have any data on the fluidity of such types of membranes containing flavors, we believe that fluidity is a useful parameter. We started looking for another useful shape parameter in addition to cell size using the Calmorph software which shape analyzing software of yeast [37]. We believe that cell shape based on the physicochemical properties of membranes is due to their components, such as flavors. Furthermore, EC-rich yeast is known to lack the fatty acid synthase gene [6]. This gene has the role of prolonging lipids; the effects would change the shape of the yeast cell. Recently shape changes using parameter analysis was studied in genome-edited yeast [38].

## 4. Conclusions

In this study, we observed changes in the size and membrane fluidity of biomimetic membranes by adding different flavors and their precursors. First, we found that the vesicles were largest when they contained 50% 1,2-dioleoyl-sn-glycero-3-phosphocholine (DOPC) with 25% EC and 25% CA. Furthermore, we found that the diameter of the EC and CA-containing vesicles was the same as the vesicles with 50% DOPC and 25% of IA and 25% of IAA. Moreover, we found that GP, as an indicator of membrane fluidity, was significantly larger for vesicles containing both 25% EC and 25% CA and 25% IA and 25% IAA though not significant compared with the other lipid vesicles. These data suggest that the diameter of yeast cells may correspond to flavor compound production from precursors in terms of membrane fluidity. Our results not only enhance our understanding of life processes but also of brewing biotechnology, including facilitating the search for yeast strains that have high flavor productivities.

## References

[1] Ichikawa E., Hosokawa N., Hata Y., Abe Y., Suginami K., Imayasu S. (1991). Breeding of a Sake yeast with improved ethyl hexanoate productivity. Agric. Biol. Chem..

[2] Ichikawa E. (1993). Sake yeast with improved ethyl caproate productivity. NihonJozogakkaishi.

[3] Akita O. (1992). Breeding of Sake yeast producing a large quantity of aroma. NihonJozogakkaishi.

[4] Verstrepen K.J., Derdelinckx G., Dufour J.P., Winderickx J., Thevelein J.M., Pretorius I.S., Delvaux F.R. (2003). Flavor-active esters: Adding fruitiness to beer. J. Biosci. Bioeng..

[5] Yoda T., Ogura A., Saito T. (2020). Influence of ethyl caproate on the size of lipid vesicles and yeast cells. Biomimetics.

[6] Akada R., Matsuo K., Aritomi K., Nishizawa Y. (1999). Construction of recombinant Sake yeast containing a dominant FAS2 mutation without extraneous sequences by a two-step gene replacement protocol. J. Biosci. Bioeng..

[7] Tsutsumi H. (2011). Seishu kōbo no kōki seisei no kenkyū (means, study on aroma production of sake yeast, in Japanese). Seibutsu-Koggakaishi.

[8] Ashida S., Ichdcawa E., Suginami K., Imayasu S. (1987). Isolation and application of mutants producing sufficient isoamyl acetate, a Sake flavor component. Agric. Biol. Chem..

[9] Mason A.B., Dufour J. (2000). Alcohol acetyltransferases and the significance of ester synthesis in yeast. Yeast.

[10] Ochiai N., Sasamoto K., Takino M., Yamashita S., Daishima S., Heiden A., Hoffman A. (2001). Determination of trace amounts of off-flavor compounds in drinking water by stir bar sorptive extraction and thermal desorption GC-MS. Analyst.

[11] Mansure J.J., Panek A.D., Crowe L.M., Crowe J.H. (1994). Trehalose inhibits ethanol effects on intact yeast cells and liposomes. Biochim. Biophys. Acta.

[12] Vanegas J.M., Contreras M.F., Faller R., Longo M.L. (2012). Role of unsaturated lipid and ergosterol in ethanol tolerance of model yeast biomembranes. Biophys. J..

[13] Sharma N., Phan H.T.T., Yoda T., Shimokawa N., Vestergaard M.C., Takagi M. (2019). Effects of capsaicin on biomimetic membranes. Biomimetics.

[14] Yoda T., Vestergaard M.C., Akazawa-Ogawa Y., Yoshida Y., Hamada T., Takagi M. (2010). Dynamic response of a cholesterol-containing model membrane to oxidative stress. Chem. Lett..

[15] Vestergaard M.C., Yoda T., Hamada T., Akazawa Y., Yoshida Y., Takagi M. (2011). The effect of oxycholesterols on thermo-induced membrane dynamic. Biochim. Biophys. Acta.

[16] Yoda T., Vestergaard M.C., Hamada T., Le P.T.M., Takagi M. (2012). Thermo-induced vesicular dynamics of membranes containing cholesterol derivatives. Lipids.

[17] Dhingra S., Morita M., Yoda T., Vestergaard M.C., Hamada T., Takagi M. (2011). Dynamic transformation of a cell-sized liposome containing ganglioside. 2011 Int. Symp. Micro-NanoMechatron. Hum. Sci..

[18] Phan H.T.T., Hata T., Morita M., Yoda T., Hamada T., Vestergaard M.C., Takagi M. (2013). The effect of oxysterols on the interaction of Alzheimer’s amyloid beta with model membranes. Biochim. Biophys. Acta Biomembr..

[19] Dhingra S., Morita M., Yoda T., Vestergaard M.C., Hamada T., Takagi M. (2013). Dynamic morphological changes induced by GM1 and protein interactions on the surface of cell-sized liposomes. Materials.

[20] Chahal B., Vestergaard M.C., Yoda T., Morita M., Takagi M. (2012). Structure-dependent membrane interaction and bioactivity of flavonoids with lipid bilayers. 2012 Int. Symp. Micro-NanoMechatron. Hum. Sci..

[21] Phan H.T.T., Yoda T., Chahal B., Morita M., Takagi M., Vestergaard M.C. (2014). Structure-dependent interactions of polyphenols with a biomimetic membrane system. Biochim. Biophys. Acta Biomembr..

[22] Ito H., Yamanaka T., Kato S., Hamada T., Takagi M., Ichikawa M., Yoshikawa K. (2013). Dynamical formation of lipid bilayer vesicles from lipid-coated droplets across a planar monolayer at an oil/water interface. Soft Matter.

[23] Ohno M., Hamada T., Takiguchi K., Homma M. (2009). Dynamic behaviors of giant liposomes at desired osmotic pressures. Langmuir.

[24] Sezgin E., Kaiser H., Baumgart T., Schwille P., Simons K., Levental I. (2012). Elucidating membrane structure and protein behavior using giant plasma membrane vesicles. Nat. Protoc..

[25] Sezgin E., Gutmann T., Buhl T., Dirkx R., Grzybek M., Coskun Ü., Solimena M., Simons K., Levental I., Schwille P. (2015). Adaptive lipid packing and bioactivity in membrane domains. PLoS ONE.

[26] Sugahara K., Shimokawa N., Takagi M. (2015). Destabilization of phase-separated structures in local anesthetic-containing model biomembranes. Chem. Lett..

[27] Official Webpage of Aomori Prefectural Industrial Technology Research Center, Hirosaki Industrial Research Institute. https://www.aomoriitc.or.jp/soshiki/kougyou_hirosaki/hirokoken/seisyukoubo.html.

[28] Iwama N. (2002). Development of Sake yeast for Ginjo-Shu brewing with use of Hanaomoi which is rice for Sake brewing. Aomori Prefectural Industrial Technology Research Center’s Report 2002.

[29] Official Webpage of Gekkeikan. http://www.gekkeikan.co.jp/RD/sake/sake05/.

[30] Chan L.L., Lyettefi E.J., Pirani A., Smith T., Qiu J., Lin B. (2011). Direct concentration and viability measurement of yeast in corn mash using a novel imaging cytometry method. J. Ind. Microbiol. Biotechnol..

[31] Arisawa K., Mitsudome H., Yoshida K., Sugimoto S., Ishikawa T., Fujiwara Y., Ichi I. (2016). Saturated fatty acid in the phospholipid monolayer contributes to the formation of large lipid droplets. Biochem. Biophys. Res. Commun..

[32] Hamada T., Miura Y., Komatsu K., Kishimoto Y., Vestergaard M., Takag M. (2008). Construction of asymmetric cell-sized lipid vesicles from lipid-coated water-in-oil micro-droplets. J. Phys. Chem. B.

[33] Van der Rest M.E., Kamminga A.H., Nakano A., Anraku Y., Poolman B., Konigs W.N. (1995). The plasma membrane of Saccharomyces cerevisiae: Structure, function, and biogenesis. Microbiol. Rev..

[34] Utsunomiya H., Yamada O., Hashiguchi T. (2000). Analysis of free fatty acids, higher alcohols and esters in *Ginfyo-shu* produced in the northern part of Kyushu. J. Brew. Soc. Jpn..

[35] Lubbers S., Charpentier C., Feuillat M., Voilley A. (1994). Influence of yeast walls on the behavior of aroma compounds in a model wine. Am. J. Enol. Vitic..

[36] Klose C., Ejsing C.S., García-Sáez A.J., Kaiser H., Sampaio J.L., Surma M.A., Shevchenko A., Schwille P., Simons K. (2010). Yeast lipids can phase-separate into micrometer-scale membrane domains. J. Biol. Chem..

[37] Ohya Y., Sese J., Yukawa M., Sano F., Nakatani Y., Saito T.L., Saka A., Fukuda T., Ishihara S., Oka S. (2005). High-dimensional and large-scale phenotyping of yeast mutants. Proc. Natl. Acad. Sci. USA.

[38] Ohnuki S., Kashima M., Yamada T., Ghanegolmohammadi F., Zhou Y., Goshima T., Maruyama J.I., Kitamoto K., Hirata D., Akao T. (2019). Genome editing to generate nonfoam-forming sake yeast strains. Biosci. Biotechnol. Biochem..

